# Effectiveness of an Internet Intervention for Family Caregivers of People with Dementia: Results of a Randomized Controlled Trial

**DOI:** 10.1371/journal.pone.0116622

**Published:** 2015-02-13

**Authors:** Marco M. Blom, Steven H. Zarit, Rob B. M. Groot Zwaaftink, Pim Cuijpers, Anne Margriet Pot

**Affiliations:** 1 Dutch Alzheimer’s Society, Department Scientific Research, Amersfoort, The Netherlands; 2 The Pennsylvania State University, Department of Human Development and Family Studies, University Park, Pennsylvania, United States of America; 3 Gerontology Institute, University College of Health Science, Jönköping, Sweden; 4 VU University, Department of Clinical Psychology, EMGO+, Amsterdam, The Netherlands; 5 Netherlands Institute of Mental Health and Addiction (Trimbos-institute), Program on Aging, Utrecht, The Netherlands; 6 University of Queensland, School of Psychology, Brisbane, Australia; The National Institute for Health Innovation, NEW ZEALAND

## Abstract

**Background:**

The World Health Organization stresses the importance of accessible and (cost)effective caregiver support, given the expected increase in the number of people with dementia and the detrimental impact on the mental health of family caregivers.

**Methods:**

This study assessed the effectiveness of the Internet intervention ‘Mastery over Dementia’. In a RCT, 251 caregivers, of whom six were lost at baseline, were randomly assigned to two groups. Caregivers in the experimental group (N = 149) were compared to caregivers who received a minimal intervention consisting of e-bulletins (N = 96). Outcomes were symptoms of depression (Center for Epidemiologic Studies Depression Scale: CES-D) and anxiety (Hospital Anxiety and Depression Scale: HADS-A). All data were collected via the Internet, and an intention-to-treat analysis was carried out.

**Results:**

Almost all caregivers were spouses or children (in-law). They were predominantly female and lived with the care recipient in the same household. Age of the caregivers varied from 26 to 87 years. Level of education varied from primary school to university, with almost half of them holding a bachelor’s degree or higher. Regression analyses showed that caregivers in the experimental group showed significantly lower symptoms of depression (p = .034) and anxiety (p = .007) post intervention after adjustment for baseline differences in the primary outcome scores and the functional status of the patients with dementia. Effect sizes were moderate for symptoms of anxiety (.48) and small for depressive symptoms (.26).

**Conclusions:**

The Internet course ‘Mastery over Dementia’ offers an effective treatment for family caregivers of people with dementia reducing symptoms of depression and anxiety. The results of this study justify further development of Internet interventions for family caregivers of people with dementia and suggest that such interventions are promising for keeping support for family caregivers accessible and affordable. The findings are even more promising because future generations of family caregivers will be more familiar with the Internet.

**Trial Registration:**

Dutch Trial Register NTR-2051 www.trialregister.nl/trialreg/admin/rctview.asp?TC=2051

## Introduction

Dementia is a worldwide healthcare challenge. According to estimations of Alzheimer Disease International, there were approximately 35.6 million people with dementia in 2010, a number that will nearly double to 65.7 million by 2030 and quadruple to 115.4 million by 2050 [1]. Most of the people with dementia—and in non-Western countries, almost all of them—live at home with the help of spouses, children, or others [1]. Providing care to a family member with dementia, however, is often a stressful experience that can erode the mental and physical health of the caregiver. Caregivers not only develop feelings of burden but also show higher levels of psychiatric symptoms, depressive and anxiety disorders, poorer immune function, and even a higher death risk compared to non-caregivers or the general population [2–6]. Therefore, regarding the important role of family caregivers for people with dementia, the World Health Organization stresses the importance of caregiver support [1].

The use of the Internet is rapidly increasing worldwide, including among older adults, and has the potential to be an effective way of delivering interventions to support family caregivers of people with dementia throughout the caregiving process [7]. Several recent meta-analyses of Randomized Controlled Trials (RCTs) on Internet interventions for younger target groups with symptoms of depression or anxiety have shown that these interventions have the potential to be (cost)effective [8–12]. From the perspective of family caregivers themselves, Internet support may have several advantages compared to face-to-face support. People can participate in an Internet course at the time that is most suitable for them; they do not have to travel to a health care professional, which saves time; and Internet support may be easier for them to accept because of the stigma associated with seeking help from a professional (mental) health care provider [13]. However, to date, results from RCTs on Internet interventions to reduce the psychiatric symptoms of caregivers of people with dementia are still scarce [7,14].

We undertook an RCT to study the effectiveness of an innovative guided self-help Internet course ‘Mastery over Dementia’ (MoD) which is designed to reduce caregivers’ symptoms of depression and anxiety [15]. The development of MoD was based on the results of meta-analyses and systematic reviews that identified effective face-to-face interventions to diminish caregivers’ psychiatric symptoms [16–18]. These techniques include a combination of psycho-education with active participation of the caregiver, management of behavioral problems, teaching coping strategies, cognitive behavioral therapy (cognitive reframing: changing non-helpful into helpful thoughts), and increasing social support. Beneficial effects of an Internet intervention for caregivers may have far-reaching consequences by making treatment much more accessible and affordable for caregivers of people with dementia around the world.

## Method

### Study design and participants

This RCT was carried out between 2010 and 2012 in The Netherlands. The Internet course MoD was compared to a minimal intervention consisting of e-bulletins sent by e-mail. This study was approved by a medical ethical review committee for mental health institutions. In Dutch: Medisch Ethische Toetsingcommissie in de Geestelijke Gezondheidszorg (METiGG). This committee received approval of the CCMO, the central committee for human research (METIGG, CCMO: NL27434.097.09). The study was registered in the Dutch trial register (NTR-2051, RCT-DDB) [13]. The authors confirm that all ongoing and related trials for this intervention are registered. The protocol for this trial and supporting CONSORT checklist are available as supporting information; see S1 CONSORT Checklist and S1 Protocol.

From 1 April 2010 until 31 December 2011, family caregivers of people with dementia were recruited via the website ‘Mastery over Dementia’, the monthly digital newsletter of the Alzheimer’s Society, leaflets at Alzheimer Cafe meetings (meetings for people with dementia, their caregivers and other interested people) and information letters to memory clinics and other relevant care institutes. After expressing interest in participating, caregivers were sent an information letter with more details on the study. They were asked to complete and sign a written informed consent form and return it by mail. Caregivers with at least some symptoms of depression or anxiety or feelings of burden were included (CES-D > 4 or HADS-A > 3 or a burden score of at least 6 on a scale ranging from 0 to 10). Caregivers with a high level of depression or anxiety (CES-D > 15 or HADS-A > 7) or who had suicidal thoughts (n = 34) were first evaluated by an elderly care physician for evaluation and to refer those who were in need of immediate medical treatment.

### Randomization and masking

Once informed consent was given, participants were stratified on sex and relationship to the person with dementia (spouse/other). A researcher not connected to the study used a computerized random-number generator for block randomization with variable sizes (experimental group = 3: comparison group = 2). This ratio was used because we expected a relatively high dropout rate for the experimental group based on earlier Internet studies, and we needed sufficient participants to gather information on the usability and feasibility of the new intervention. Participants did not know whether the intervention they received was the experimental or the comparison intervention. The data were all collected via the Internet with no intermediary interviewer.

### Internet course and e-bulletins

**Experimental group: Internet course Mastery over Dementia (MoD)**. The Internet course consists of 8 lessons and a booster session with the guidance of a coach monitoring the progress of participants and evaluating the homework. Each lesson has the same structure and consists of information (text material and videos), exercises, and homework, with an evaluation at the start and end of each session. The elements of the course are presented in the following order: coping with behavioral problems (problem solving); relaxation; arranging help from others; changing non-helping thoughts into helping thoughts (cognitive restructuring); and communication with others (assertiveness training) [15]. The booster session is provided a month after participants finish the eight lessons, and it provides a summary of what has been learned. After every lesson, participants sent their homework to a coach via a secure application. The coach sent electronic feedback to caregivers on their homework within three working days. The feedback had to be opened before the next lesson can be started. Participants were automatically reminded to start with a new lesson or to send in their homework if they were not active for a fixed period of time. All participants in this study received feedback from the same coach, a psychologist employed by a health care agency with additional training in cognitive behavioral therapy and experience in the field of dementia.

**Comparison group: e-bulletins**. Caregivers in the comparison group received a minimal intervention consisting of e-bulletins (digital newsletters) with practical information on providing care for someone with dementia. The bulletins were sent by email according to a fixed schedule (every 3 weeks) over nearly 6 months. The topics of the bulletin, which did not overlap with the content of MoD, were driving; holiday breaks; medication; legal affairs; activities throughout the day; help with daily routines; grieving; safety measures in the home; and possibilities for peer support. There was no contact with a coach.

### Procedures

Participants completed assessment at three time points: a baseline measurement (before the start of the intervention), a short assessment halfway through the programs (after the fourth MoD-lesson or e-bulletin), and a post-treatment assessment directly after the last lesson or e-bulletin, 5 to 6 months after baseline. All measures used were self-report and administered through the Internet. Regular automated emails were sent to all participants to alert them to complete the questionnaires.

The primary outcome was symptoms of depression. Depressive symptoms were measured using the Center for Epidemiologic Studies Depression Scale (CES-D) consisting of 20 items [19]. The total score range is 0 to 60. Symptoms of anxiety were the secondary outcome. The 7-item anxiety subscale of the Hospital Anxiety and Depression Scale was used to measure the severity of anxiety symptoms [20]. The total score ranges from 0 to 21. Demographics were obtained (sex, age, relationship to the care recipient, level of education, sharing household, duration of symptoms). Functional status of the person with dementia was measured with the 16-item IQCODE, with a total score ranging from 0 to 64 [21].

Four additional measures were used for imputation (see statistical analysis). Perceptions of distress were obtained using the Self-Perceived Pressure from Informal Care scale [22]. The SPPIC consists of 9 items, summed on the basis of dichotomized scores, with a total range from 0 to 9. The Revised Memory and Behavioral Problem Checklist (RMBPC) was used, including 24 items of caregivers’ distress related to memory and behavior problems [23]. A mean product score ranging from 0 to 16 was calculated by adding up per item the product score of frequency times the level of perceived stress indicator, divided by the number of behavioral problems. The caregivers’ sense of competence was assessed by the Short Sense of Competence Questionnaire [24]. The SSCQ consists of seven items, summed on the basis of dichotomized scores, with a total range from 0 to 7. Sense of mastery was assessed using an abbreviated five-item version of the Pearlin Mastery Scale [25]. Items are summed for a total mastery score with a range from 0 to 20.

### Statistical analysis

For intention-to-treat analysis, missing data due to dropout after baseline were imputed by using demographics, the scores on primary and secondary outcome measures, and the four additional measures as predictors. The number of imputations was set to 5. There were no missing data other than those caused by dropout of participants. In the statistical analyses pooled data of the multiple imputation were used. Firstly, a regression analysis was used to test whether the treatment variable (receiving MoD versus e-bulletins) predicted the change in caregivers’ symptoms of depression and anxiety from pre-test to post-test, adjusted for baseline differences. Secondly, we calculated effect sizes for the improvement of the experimental and comparison groups by dividing the absolute difference between the post- and pre-intervention average score by the pre-intervention standard deviation. For between-group effect sizes, differences between the mean changes in both groups were divided by the average standard deviation at baseline for both groups. Effect sizes of 0.56–1.2 can be assumed to be large, effect sizes of 0.33–0.55 moderate, and effect sizes of 0–0.32 small [26]. For the analysis and multiple imputations, we used SPPS for Windows, version 20.

## Results

**Fig 1:**
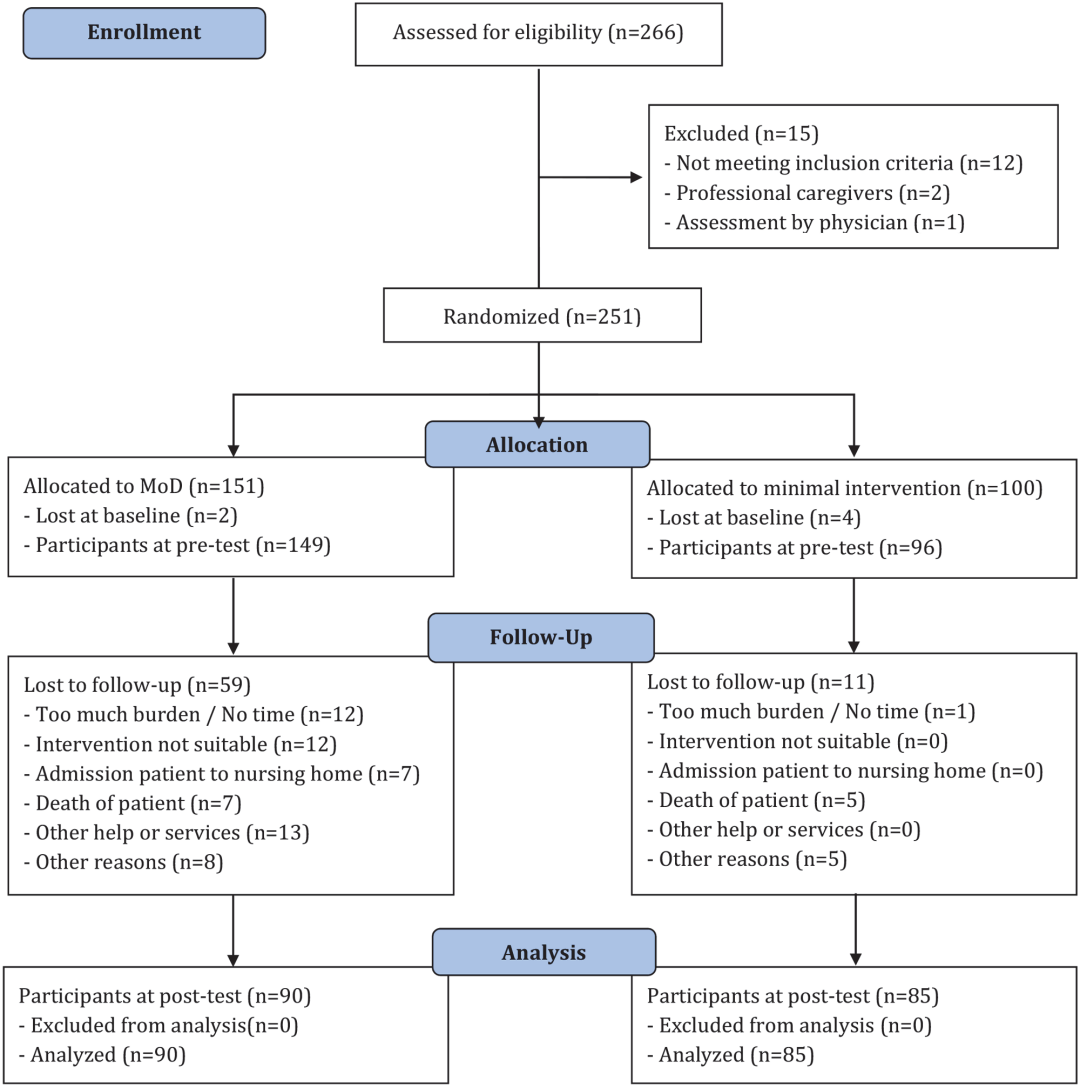
Participant flow and follow-up.

In total, 245 caregivers were enrolled in the study: 149 in the experimental group and 96 in the comparison group. Although 266 caregivers signed up for this study, 14 were excluded because they did not fulfill the inclusion criteria, one because the elderly care physician felt that immediate medical treatment was required, and six caregivers were lost at baseline because they did not respond after the randomization. Seventy caregivers (28.6%) dropped out before the end of the intervention period: 59 in the experimental group (51 in the first 3 months), and 11 in the comparison group (all between 3 and 6 months). The main reasons for dropout were ‘lack of time or energy’ (18.6%), ‘using other services’ (18.6%), ‘intervention less suitable’ (17.1%), ‘death of the care recipient’ (17.1%) or ‘admission of the care recipient to a nursing home’ (10.0%), and ‘other reasons’ (18.6%). At the end of the intervention, 175 caregivers took the post-test: 90 (60.4%) in the experimental group and 85 (88.5%) in the comparison group (Fig. 1). There were no differences found between caregivers who dropped out and those who completed the study. Within the experimental group there was one significant difference found. Caregivers who lived with the care recipient in the same household were more likely to complete the study (P = .027). Within the comparison group no differences were found.

### Sample characteristics

Almost all caregivers were spouses (58.4%) or children (in-law) (39.6%). They were predominantly female (69.4%) and lived with the care recipient in the same household (60.4%). The mean age of the caregivers was 61.2 years (range 26 to 87, SD = 12.37). Most caregivers had children (78.8%: M = 2.54, SD = 1.10). All but two held Dutch nationality (99.2%). The level of education varied from primary school to university, with 47.3 percent holding a bachelor’s degree or higher.

The mean score on the SPPIC was 7.1 (SD = 1.98), on the RMBPC-Reaction 6.5 (SD = 2.05), on the SSCQ 5.3 (SD = 1.79), and on the Mastery Scale 8.4 (SD = 3.78). The mean score on the CES-D was 17.4 (SD = 9.35). More than half of the caregivers, more specifically 131 (53.5%), had a CES-D score above cut-off (> = 16), indicating that they had a high risk of depressive disorder. On the HADS-A, the mean score was 8.1 (SD = 3.49), with 129 caregivers (52.7%) scoring above cut-off (> = 8). Statistical analysis showed no significant differences between the experimental and comparison groups at baseline (Table 1).

**Table 1:** Descriptive data on caregivers of people with dementia for both groups at baseline.

	Experimental group (MoD: n = 149)	Comparison group (E-bulletin: n = 96)	Chi / T-test	P-value
Sex (female)	104 (69.8%)	66 (68.8%)	.030	.862
Relationship (spousal)	89 (59.7%)	54 (56.3%)	.291	.589
Same household as care recipient	92 (61.7%)	56 (58.3%)	.284	.594
Dutch nationality	147 (98.7%)	96 (100%)	1.299	.254
Education: > = bachelor degree	74 (49.7%)	42 (43.8%)	.819	.365
Having children: yes	114 (76.5%)	79 (82.3%)	1.167	.280
Number of children	M = 2.57 (SD = 1.12) Range: 1 to 6	M = 2.49 (SD = 1.07) Range: 1 to 7	.475	.636
Age	M = 61.54 (SD = 11.93) Range: 33 to 87	M = 60.77 (SD = 13.07) Range: 26 to 83	.477	.634
Burden (SPPIC)	M = 7.20 (SD = 1.94) Range: 0 to 9	M = 6.95 (SD = 2.04) Range: 0 to 9	.978	.329
Distress (RMBPC-Reaction)	M = 6.32 (SD = 1.84) Range: 2.58 to 11.63	M = 6.72 (SD = 2.32) Range: 2.5 to 12.13	-1.437	.153
Competence (SSCQ)	M = 5.28 (SD = 1.78) Range: 0 to 7	M = 5.36 (SD = 1.82) Range: 0 to 7	-.352	.725
Mastery (Pearlin Mastery Scale)	M = 8.47 (SD = 3.74) Range: 0 to 18	M = 8.38 (SD = 3.86) Range: 0 to 19	.191	.848
Depressive symptoms (CES-D)	M = 17.90 (SD = 9.14) Range: 3 to 45	M = 16.61 (SD = 9.68) Range: 0 to 48	1.050	.295
Symptoms of anxiety (HADS-A)	M = 8.36 (SD = 3.36) Range: 2 to 21	M = 7.77 (SD = 3.68) Range: 1 to 17	1.297	.196

The majority of the persons with dementia were female (60.4%), lived independently (82.9%) and had a mean age of 75.9 years (range 39 to 93, SD = 9.40). Care recipients had been formally diagnosed by a doctor (86.5%) as having a dementia syndrome, with Alzheimer’s Disease as the most frequently mentioned disease (74.5%). Slightly over half of the patients (52.4%) received a diagnosis within the last 2 years. The mean total score on the IQCODE was 58.9 (SD = 5.75). Statistical analysis (Table 2) showed no significant differences between the groups on baseline measures, except that the functional status (IQCODE) of patients in the experimental group was better (lower score) than in the comparison group (P = .004). In the regression analysis adjustment was made for the difference in functional status.

**Table 2:** Descriptive data on persons with dementia for both groups at baseline.

	Experimental group (MoD: n = 149)	Comparison group (E-bulletin: n = 96)	Chi / T-test	P-value
Sex (female)	91 (61.1%)	57 (59.4%)	.070	.791
Living independently (yes)	127 (85.2%)	76 (79.2%)	1.514	.219
Formal diagnosis (yes)	125 (83.9%)	87 (90.6%)	2.270	.132
Diagnosis (Alzheimer’s)	99 (79.2%)	59 (67.8%)	.633	.426
Time since diagnosis (< 2 yr)	69 (55.2%)	42 (48.3%)	.986	.321
Age (yrs)	M = 76.36 (SD = 9.45) Range: 39 to 93	M = 75.20 (SD = 9.32) Range: 54 to 91	.941	.348
Severity of dementia (IQ-CODE)	M = 58.09 (SD = 6.42) Range: 31 to 64	M = 60.07 (SD = 4.29) Range: 47 to 64	-2.902	.004

### Effectiveness

The results showed that the treatment variable had a significant effect on the outcome scores for symptoms of depression (B = 2.208; P = .034) and anxiety (B = 1.114; P = .007), after adjustment for outcome scores at baseline and the differences found in the functional status of the people with dementia at baseline (Table 3). For the experimental group, the mean scores on the CES-D decreased from 17.89 at pre-test to 15.55 at post-test (M = 2.35; SD = 8.21), resulting in an effect size of. 26. For the comparison group, the mean scores increased from 16.61 at pre-test to 16.95 at post-test (M = -.034; SD = 7.51), resulting in an effect size of-.04. On the HADS-A, the mean scores of the caregivers in the experimental group changed from 8.36 to 6.68 (M = 2.32; SD = 3.26), resulting in an effect size of. 48, whereas caregivers’ scores in the comparison group decreased from 7.77 at pre-test to 7.30 at post-test (M = 0.47; SD = 3.41), resulting in an effect size of. 13. The differences in effect sizes between both groups are moderate for the HADS-A scores (.34) and small for the CES-D scores (.29) (Table 4).

**Table 3:** Effect of treatment condition on the change in symptoms of depression and anxiety from pre- to post measurement: T-test with pre and post scores and mean change per group and regression analysis with MoD as treatment condition.

	Experimental	Comparison	T-test	P-value	Unstandardized B-coefficient	P-value
Change in depressive symptoms	Pre: 17.89 Post: 15.55 M = 2.35 SD = 8.21	Pre: 16.61 Post: 16.95 M = -0.34 SD = 7.51	2.410	.016	2.208	.034
Change in symptoms of anxiety	Pre: 8.36 Post: 6.68 M = 1.69 SD = 3.26	Pre: 7.77 Post: 7.30 M = 0.47 SD = 3.41	2.679	.008	1.114	.007

^1^ adjusted for baseline functional status of the people with dementia and baseline outcome measures

**Table 4:** Effect sizes (Cohen’s D) of the differences between post-test and pre-test scores for symptoms of depression and anxiety.

Effect sizes	Experimental group (MoD: n = 149)	Comparison group (E-bulletin: n = 96)	Difference in effect sizes between groups
Depressive symptoms (CES-D)	.26	-.04	.29
Symptoms of anxiety (HADS-A)	.48	.13	.34

Statistical analysis with T-test on non-imputed data of those participants who completed the intervention, respectively 90 family caregivers in the experimental group and 85 family caregivers in the comparison group, was conducted. No differences were found at baseline between both groups on CES-D (T = 1.372; P = .172) or HADS-A (T = 0.908; P = .365). For the experimental group, the mean scores on the CES-D decreased from 18.23 at pre-test to 14.92 at post-test (M = 3.31; SD = 7.92). Mean score on the HADS-A changed from 8.24 to 6.23 (M = 2.01; SD = 2.97). For caregivers in the comparison group, mean scores on CES-D changed from 16.27 to 16.85 (M = -0.58; SD = 8.13). Scores on the HADS-A decreased from 7.75 to 7.25 (M = 0.51; SD = 3.39). T-tests between both groups showed significant differences on both the CES-D (T = 3.204; P = .002) and HADS-A (T = 3.125; P = .0023).

## Discussion

This first randomized controlled trial on the effectiveness of a guided self-help Internet therapy for family caregivers of people with dementia, ‘Mastery over Dementia’ (MoD), showed that caregivers’ symptoms of depression and anxiety were significantly reduced after receiving MoD, compared to a minimal intervention in which caregivers received digital newsletters by e-mail. The effect sizes for caregivers who received the Internet course were found to be moderate for symptoms of anxiety (.48) and small for symptoms of depression (.26), as were the differences in effect sizes between the experimental and the comparison group (.34 and. 29, respectively). This study also showed that even in an older age group of caregivers above 65 years, who, to a large extent, have not grown up with the Internet, participation in an Internet course to reduce psychological symptoms is feasible. To date, most participants in randomized controlled trials to decrease psychological distress via Internet courses have not been over 65 years [27].

The results of this study are promising and in line with the results found in a recent Cochrane meta-analysis on cognitive reframing for family caregivers of people with dementia to diminish caregivers’ psychological distress. The study showed Standardized Mean Differences of-.21 for symptoms of anxiety and-.66 for symptoms of depression. However, after removing one study to reduce heterogeneity between studies, the SMD was found to be-.24 [28]. Thus, Internet interventions such as ‘Mastery over Dementia’ might augment face-to-face interventions for caregivers, which holds potential in light of the anticipated growth in numbers of people with dementia in the next decades and the increasing importance of the role of family caregivers, especially in high-income countries. This is especially important because caregivers of people with dementia may develop high levels of psychological distress, as confirmed by the current study’s findings that the majority of the participants had clinically relevant levels of depression and anxiety symptoms. This study has several limitations. Firstly, almost three in ten caregivers resigned from the study, especially those who participated in the Internet course (40% versus 11% in the e-bulletin group). Dropout is a common problem in psychotherapy research and even more so in Internet therapy research, where mean dropout rates of 35% have been found, ranging from 2% to 83% [29]. The dropout rate may be somewhat higher due to the lengthy and time-intensive character of the Internet course MoD [30]. In the statistical analysis, we used an intention to treat principle to avoid effects of dropout. However, there is still the possibility of remaining bias due to the reasons for dropout we could trace in this study. Statistical analysis on non-imputed data showed similar results. Secondly, differences between experimental group and comparison group in content of the intervention and the methods used to deliver the intervention may make the findings less robust. However, in most studies in this field caregivers in control groups often receive care as usual, instead of care as usual plus an extra minimal intervention. Thirdly, participants were better educated than the general population (http://www.trendsinbeeld.minocw.nl/grafieken/3_1_2_31.php). Therefore, it is uncertain whether the results can be generalized to the population of caregivers as a whole. Fourthly, because we provided information on different topics in the experimental and comparison group, we cannot compare the different ways in which the information was provided in both groups. Fifthly, the coach who was involved in the Internet course was instructed to provide feedback on the exercises only; however, we did not formally check the adherence to this instruction. Further research is needed to clarify the necessity of the feedback of a coach. A completely self-help Internet course would reduce the costs of providing support for the increasing number of family caregivers for people with dementia and would increase its accessibility. However, a coach might help people who are struggling with the intervention to stick to the right track or encourage them to focus and continue. Ambiguous results regarding the role of a coach or therapist in Internet inventions have been found. Some studies showed that guided self-help is more effective than self-help without guidance [31]. Other studies revealed that Internet therapy with no or brief therapist support has a beneficial impact comparable to face-to-face therapy [11].

Although this RCT showed that ‘Mastery over Dementia’ is effective in treating symptoms of depression and anxiety in family caregivers of people with dementia as a whole, Internet support or therapy will not suit every caregiver: some will benefit, others will not. Further research is needed to answer the question of which Internet interventions or intervention components are feasible and effective for which particular caregivers, to improve treatment response. An earlier study on the effectiveness of a multimedia support program delivered over the Internet to employed family caregivers showed improvements on depression, anxiety and caregiver stress. The intervention provided both text material and videos that modeled positive caregiving strategies. Participants were not in contact with a coach [14]. A recent article showed positive effects on perceived stress for caregivers who followed an Internet-based program that focused on teaching coping skills to caregivers for stress management. The intervention was adapted from a psycho-educational program that was effective when delivered in a small group format requiring face-to-face contact [32]. However, no significant changes were found with respect to depressive symptoms, expressed bother in relation to problem behavior or perceived quality of life. In addition, more information is needed on the (cost) effectiveness of Internet courses in the long run to keep the support for family caregivers accessible and affordable, thus serving people with dementia as well.

## Supporting Information

S1 CONSORT Checklist Consort Checklist. (DOC)

S1 Protocol Article published in *BMC Psychiatry* 2013: 13–17. (PDF)
